# Endoscopic ultrasound-guided pancreatic duct drainage with a two-step puncture technique for a non-dilated pancreatic duct

**DOI:** 10.1055/a-2277-0836

**Published:** 2024-03-14

**Authors:** Shunsuke Horitani, Masaaki Shimatani, Masataka Kano, Toshiyuki Mitsuyama, Tsukasa Ikeura, Shuntaro Mukai, Takao Itoi

**Affiliations:** 1 50196 Division of Gastroenterology and Hepatology, Third Department of Internal Medicine, Kansai Medical University Medical Center, Osaka, Japan; 2 50196 Divison of Gastroenterology and Hepatology, Third Department of Internal Medicine, Kansai Medical University Medical Center, Osaka, Japan; 3 50196 Division of Gastroenterology and Hepatology, Third Department of Internal Medicine, Kansai Medical University Medical Center, Osaka, Japan; 4 Division of Gastroenterology and Hepatology, Third Department of Internal Medicine, Kansai Medical University, Hirakata, Japan; 5 13112 Department of Gastroenterology and Hepatology, Tokyo Medical University, Tokyo, Japan

Endoscopic ultrasound-guided pancreatic duct drainage (EUS-PD) has emerged as an option in patients with failure of standard transpapillary endoscopic retrograde access to the pancreatic duct (PD) or surgically altered anatomy 1 . The ductal pressure of a non-dilated PD is often low and the duct can be easily compressed by the tip of the needle, thus requiring a technical tip to handle this situation 2 . We describe successful EUS-PD with a two-step puncture technique for a non-dilated PD after pancreaticoduodenectomy as a result of solid pseudopapillary neoplasm ( Video 1 ).

Endoscopic ultrasound-guided pancreatic duct drainage with two-step puncture technique for a non-dilated pancreatic duct was achieved. This method is effective for draining a non-dilated pancreatic duct.Video 1

An 18-year-old woman was referred to our hospital because of gradual PD dilatation after pancreaticoduodenectomy. A double-balloon endoscopy was performed but failed to detect the pancreatic-jejunal anastomosis. Subsequently, EUS-PD was performed. A convex ultrasound gastrovideoscope (GF-UCT260; Olympus, Tokyo, Japan) was used to puncture from the gastric stomach to the caudal PD with a 22-gauge EUS-guided fine-needle (EZ Shot 3 Plus, Olympus). EUS and fluoroscopy both revealed a 2-mm non-dilated PD ( Fig. 1 **a** , **b** ), but an 0.018-inch guidewire (Fielder, Olympus) could not be advanced into the non-dilated PD. Therefore, contrast injection was continued to temporarily increase the ductal pressure and dilate the PD ( Fig. 2 ). The dilated PD facilitated subsequent re-puncture by a fine needle and allowed the guidewire to proceed into the PD ( Fig. 3 **a** , **b** ). Then the puncture tract was dilated using a 7 Fr drill dilator (Tornus ES, Olympus). After dilation of the tract, an MTW catheter (ABIS, Tokyo, Japan) was inserted into the PD. An 0.025-inch guidewire was advanced through the PJA. Then the PJA was dilated with a 3-mm diameter balloon catheter (REN, Kaneka Medix, Osaka, Japan). Finally, a 15-cm 7 Fr plastic stent (TYPE-IT; Gadelius Medical K.K., Tokyo, Japan) was placed from the jejunum to the stomach through the PD ( Fig. 4 ). No complications were observed after the procedure.

**Fig. 1:**
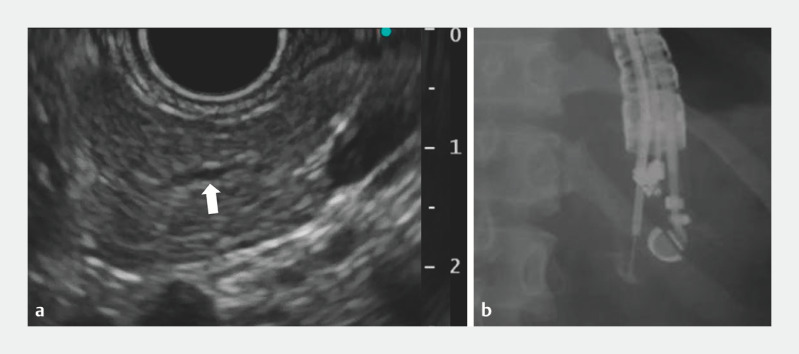
**a** Endoscopic ultrasound revealed a 2-mm non-dilated pancreatic duct (arrow). **b** Contrast injection showing pancreatogram.

**Fig. 2:**
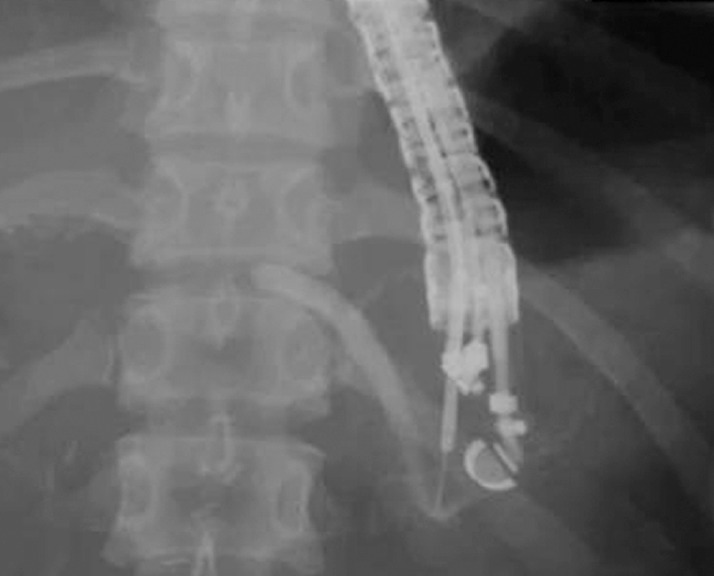
The pancreatic duct was dilated by the continuous injection of contrast medium.

**Fig. 3:**
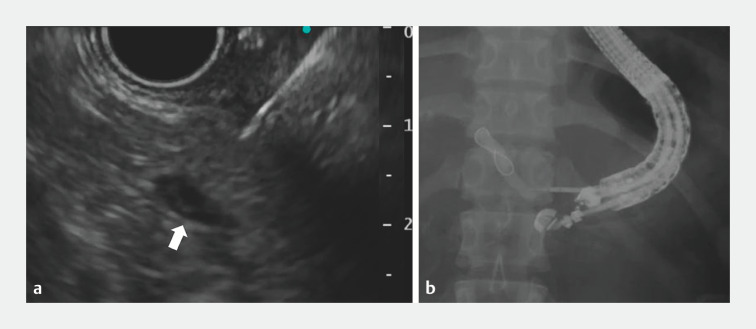
**a** The dilatation of the pancreatic duct by contrast injection (arrow) facilitated re-puncture by the fine-needle. **b** The guidewire could be advanced into the pancreatic duct

**Fig. 4:**
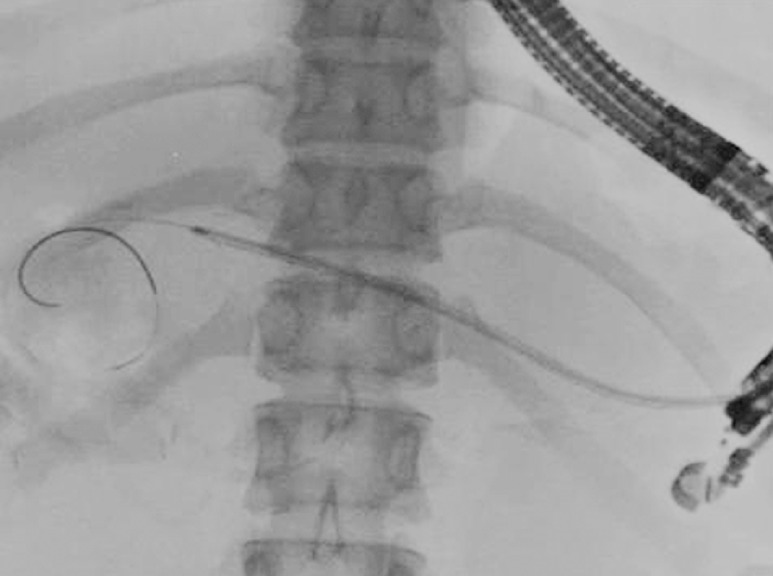
A plastic stent was placed from the jejunum to the stomach through the pancreatic duct.

EUS-PD with a two-step puncture technique is effective for draining a non-dilated PD.

Endoscopy_UCTN_Code_TTT_1AS_2AI

## Notes

Endoscopy E-Videos https://eref.thieme.de/e-videos
*E-Videos* is an open access online section of the journal *Endoscopy* , reporting on interesting cases and new techniques in gastroenterological endoscopy. All papers include a high-quality video and are published with a Creative Commons CC-BY license. Endoscopy E-Videos qualify for HINARI discounts and waivers and eligibility is automatically checked during the submission process. We grant 100% waivers to articles whose corresponding authors are based in Group A countries and 50% waivers to those who are based in Group B countries as classified by Research4Life (see: https://www.research4life.org/access/eligibility/ ). This section has its own submission website at https://mc.manuscriptcentral.com/e-videos .
